# Dietary and Physical Activity Behaviors Among High School Students — Youth Risk Behavior Survey, United States, 2019

**DOI:** 10.15585/mmwr.su6901a8

**Published:** 2020-08-21

**Authors:** Caitlin L. Merlo, Sherry Everett Jones, Shannon L. Michael, Tiffany J. Chen, Sarah A. Sliwa, Seung Hee Lee, Nancy D. Brener, Sarah M. Lee, Sohyun Park

**Affiliations:** ^1^Division of Population Health, National Center for Chronic Disease Prevention and Health Promotion, CDC; ^2^Division of Adolescent and School Health, National Center for HIV/AIDS, Viral Hepatitis, STD, and TB Prevention, CDC; ^3^Division of Nutrition, Physical Activity, and Obesity, National Center for Chronic Disease Prevention and Health Promotion, CDC

## Abstract

Establishing healthy dietary and physical activity patterns among youths is an important public health strategy for improving health and preventing chronic diseases; however, few adolescents meet U.S. government recommendations for dietary or physical activity behaviors, and disparities by sex and race/ethnicity exist. CDC analyzed data from the 2019 Youth Risk Behavior Survey to update estimates of dietary and physical activity behaviors among U.S. high school students overall and by sex and race/ethnicity. In addition, 2-year comparisons (2017 and 2019) and trends in prevalence of these behaviors during 2009–2019 were examined. In 2019, overall, during the 7 days before the survey, 41.8% of students had eaten fruit or drunk 100% fruit juices <1 time/day; 40.7% had eaten vegetables <1 time/day; and 16.7% had not eaten breakfast on all 7 days. Moreover, although 57.4% of students had played on ≥1 sports team during the 12 months before the survey, less than half of students had been physically active for ≥60 minutes/day on all 7 days (23.2%), had exercised to strengthen or tone their muscles on ≥3 days/week (49.5%), had met both aerobic and muscle-strengthening physical activity guidelines (16.5%), or had attended physical education classes on all 5 days in an average school week (25.9%). Trend data indicate limited progress in shifting dietary and physical activity behaviors. That is, with the exception of decreases in the percentage of students who had consumed soda ≥1 time/day (2009: 29.2%; 2019: 15.1%), sports drinks ≥1 time/day (2015: 13.8%; 2019: 10.6%), and <3 glasses/day of plain water (2015: 50.5%; 2019: 44.6%), high school students’ dietary and physical activity behaviors have not improved and, in certain cases, have worsened. These findings support the need for multicomponent approaches, including policy and environmental changes, and opportunities for adolescents to learn about and practice making healthy choices.

## Introduction

Nutrition and physical activity are important for optimal growth and development and chronic disease prevention. Approximately half of U.S. adults have a chronic disease that is related to inadequate diet quality and physical activity, including type 2 diabetes, cardiovascular diseases, or obesity (*1*). Such diseases can affect productivity and quality of life and contribute to high health-care costs. Dietary and physical activity behaviors develop during childhood and can create a trajectory that continues into adulthood (*1**,**2*). Establishing healthy dietary and physical activity behaviors early in life is a vital public health strategy for promoting lifelong physical health.

The U.S. government establishes recommendations for healthy dietary and physical activity patterns for persons of different ages, including adolescents. The *Dietary Guidelines for Americans 2015–2020* outlines key recommendations for following a healthy eating pattern within calorie levels that are appropriate for a person’s age, sex, height, weight, and physical activity level (*1*). Recommendations include eating a variety of vegetables, fruits, and whole grains, and limiting sodium, added sugars, and saturated and *trans* fats.

The *Physical Activity Guidelines for Americans*, 2nd Edition, recommends that children and adolescents ages 6–17 years engage in ≥60 minutes of moderate-to-vigorous physical activity daily (*2*). Most of this daily physical activity should be aerobic activity, whereas muscle- and bone-strengthening physical activity should each be done ≥3 days each week (*2*).

Previous data indicate that most adolescents are not meeting recommendations for healthy eating (*1*) or physical activity (*3*), which increases the risk for chronic diseases later in life. In addition, disparities by sex and race/ethnicity exist (*4*). To update national estimates of dietary and physical activity behaviors among U.S. high school students overall and by sex and race/ethnicity and to determine how these behaviors have changed over time, CDC analyzed data from the 2019 Youth Risk Behavior Survey (YRBS) and examined trends in prevalence of these behaviors during the previous 10 years. Public health and school health researchers and practitioners can use these findings to inform policies and practices that support healthy eating and physical activity among adolescents. 

## Methods

### Data Source

This report includes data from the 2009–2019 cycles of the YRBS, a cross-sectional, school-based survey conducted biennially since 1991. Each survey year, CDC collects data from a nationally representative sample of public and private school students in grades 9–12 in the 50 U.S. states and the District of Columbia. Additional information about YRBS sampling, data collection, response rates, and processing is available in the overview report of this supplement (*5*). The prevalence estimates for all physical activity, nutrition, and body weight questions for the overall study population and by sex, race/ethnicity, grade, and sexual orientation are available at https://nccd.cdc.gov/youthonline/App/Default.aspx. The full YRBS questionnaire is available at https://www.cdc.gov/healthyyouth/data/yrbs/pdf/2019/2019_YRBS-National-HS-Questionnaire.pdf.

### Measures

The student demographic characteristics analyzed included sex (female or male) and race/ethnicity. Students were classified into four racial/ethnic categories: non-Hispanic white (white); non-Hispanic black (black); Hispanic or Latino of any race (Hispanic); and other or multiple races. The numbers of students in the other or multiple racial/ethnic groups were too small for meaningful analysis; therefore, findings for those groups are not presented; however, the corresponding data remain in the analytic sample. This analysis included six dietary variables and five physical activity variables (Table 1). The dietary variables included the following: during the 7 days before the survey, had eaten fruit or drunk 100% fruit juices <1 time/day, had eaten vegetables <1 time/day, had not eaten breakfast on all 7 days, had drunk soda or pop ≥1 time/day (not counting diet soda or diet pop), had drunk a sports drink ≥1 time/day, and had drunk <3 glasses/day of plain water. The physical activity variables included the following: during the 7 days before the survey, had been physically active for a total of ≥60 minutes/day on all 7 days, had exercised to strengthen or tone muscles on ≥3 days, had met both aerobic and muscle-strengthening physical activity guidelines (defined as being physically active for a total of ≥60 minutes/day on all 7 days and doing exercises to strengthen or tone muscles on ≥3 days), had attended physical education classes on all 5 days in an average school week, and had played on ≥1 sports team during the 12 months before the survey. 

**TABLE 1:** Question wording and details for included dietary and physical activity behavior variables — Youth Risk Behavior Survey, United States, 2019

Variable	Question	Response options	Years of data available for 10-year trend analysis	Coding for analysis
Dietary behaviors				
Ate fruit or drank 100% fruit juices <1 time/day	During the past 7 days, how many times did you . . . • drink 100% fruit juices such as orange juice, apple juice, or grape juice? (Do not count punch, Kool-Aid, sports drinks, or other fruit-flavored drinks.) • eat fruit? (Do not count fruit juice.)	I did not [drink 100% fruit juice]/[eat fruit] during the past 7 days, 1–3 times during the past 7 days, 4–6 times during the past 7 days,1 time/day, 2 times/day, 3 times/day, or ≥4 times/day	2009–2019	<1 time/day versus ≥1 time/day
Ate vegetables <1 time/day	During the past 7 days, how many times did you eat . . . • green salad? • potatoes? (Do not count French fries, fried potatoes, or potato chips.) • carrots? • other vegetables? (Do not count green salad, potatoes, or carrots.)	I did not eat [green salad]/[potatoes]/[carrots]/[other vegetables] during the past 7 days, 1–3 times during the past 7 days, 4–6 times during the past 7 days, 1 time/day, 2 times/day, 3 times/day, or ≥4 times/day	2009–2019	<1 time/day versus ≥1 time/day
Did not eat breakfast on all 7 days	During the past 7 days, on how many days did you eat breakfast?	0 days, 1 day, 2 days, 3 days, 4 days, 5 days, 6 days, or 7 days	2011–2019	<7 days versus 7 days
Drank soda or pop ≥1 time/day	During the past 7 days, how many times did you drink a can, bottle, or glass of soda or pop, such as Coke, Pepsi, or Sprite? (Do not count diet soda or diet pop.)	I did not drink soda or pop during the past 7 days, 1–3 times during the past 7 days, 4–6 times during the past 7 days, 1 time/day, 2 times/day, 3 times/day, or ≥4 times/day	2009–2019	≥1 time/day versus <1 time/day
Drank a sports drink ≥1 time/day	During the past 7 days, how many times did you drink a can, bottle, or glass of a sports drink, such as Gatorade or Powerade? (Do not count low-calorie sports drinks such as Propel or G2.)	I did not drink sports drinks during the past 7 days, 1–3 times during the past 7 days, 4–6 times during the past 7 days, 1 time/day, 2 times/day, 3 times/day, or ≥4 times/day	2015–2019	≥1 time/day versus <1 time/day
Drank <3 glasses/day of plain water	During the past 7 days, how many times did you drink a bottle or glass of plain water? (Count tap, bottled, and unflavored sparkling water.)	I did not drink water during the past 7 days, 1–3 times during the past 7 days, 4–6 times during the past 7 days, 1 time per day, 2 times per day, 3 times/day, or ≥4 times/day	2015–2019	≥3 times/day versus <3 times/day
**Physical activity behaviors**				
Were physically active for a total of ≥60 minutes/day on all 7 days	During the past 7 days, on how many days were you physically active for a total of at least 60 minutes per day? (Add up all the time you spent in any kind of physical activity that increased your heart rate and made you breathe hard some of the time.)	0 days, 1 day, 2 days, 3 days, 4 days, 5 days, 6 days, or 7 days	2011–2019	7 days versus <7 days
Did exercises to strengthen or tone muscles on ≥3 days	During the past 7 days, on how many days did you do exercises to strengthen or tone your muscles, such as push-ups, sit-ups, or weightlifting?	0 days, 1 day, 2 days, 3 days, 4 days, 5 days, 6 days, or 7 days	2011–2019	≥3 days versus <3 days
Met both aerobic and muscle-strengthening physical activity guidelines	[See “were physically active for a total of ≥60 minutes/day on all 7 days” and “did exercises to strengthen or tone muscles on ≥3 days.”]	Not applicable	2011–2019	Physically active for ≥60 minutes/day on all 7 days *and* did exercises to strengthen or tone muscles on ≥3 days versus physically active for <60 minutes/day on all 7 days *or* did exercises to strengthen or tone muscles on <3 days
Attended physical education classes on all 5 days	In an average week when you are in school, on how many days do you go to physical education (PE) classes?	0 days, 1 day, 2 days, 3 days, 4 days, or 5 days	2009–2019	≥5 days versus <5 days
Played on ≥1 sports team	During the past 12 months, on how many sports teams did you play? (Count any teams run by your school or community groups.)	0 teams, 1 team, 2 teams, or ≥3 teams	2009–2019	≥1 team versus <1 team

### Analysis

Prevalence estimates and 95% confidence intervals for each 2019 dietary and physical activity behavior were calculated overall and for each sex and racial/ethnic group. Statistically significant pairwise differences by sex and race/ethnicity were determined by *t*-tests. In addition, prevalence of each dietary and physical activity behavior was compared for 2017 with 2019 by using *t*-tests. Differences between prevalence estimates were considered statistically significant if the *t*-test p value was <0.05. 

To identify 10-year temporal trends, logistic regression analyses were used to model linear and quadratic time effects while controlling for sex, grade (9, 10, 11, and 12), and racial/ethnic changes over time (*6*). All variables had data available for 2009–2019, except for did not eat breakfast on all 7 days; were physically active for a total of ≥60 minutes/day on all 7 days; did exercises to strengthen or tone muscles on ≥3 days and met both aerobic and muscle-strengthening physical activity guidelines, which had data for 2011–2019 only; and drank a sports drink ≥1 time/day and drank <3 glasses/day of plain water, which had data for 2015–2019 only. Additional information about the methods used to conduct YRBS trend analyses are provided in the overview report of this supplement (*5*).

## Results

### Dietary Behaviors

#### Overall

In 2019, nationwide, 41.8% of students had eaten fruit or drunk 100% fruit juices <1 time/day; 40.7% had eaten vegetables <1 time/day; 16.7% had not eaten breakfast on all 7 days; 15.1% had drunk sugar-sweetened soda or pop ≥1 time/day (not counting diet soda or diet pop); 10.6% had drunk a sports drink ≥1 time/day; and 44.6% had drunk <3 glasses/day of plain water (Table 2). A higher percentage of male students than female students had drunk sugar-sweetened soda or pop ≥1 time/day (18.2% versus 11.7%) and had drunk a sports drink ≥1 time/day (14.0% versus 7.1%). A higher percentage of black students than white and Hispanic students had eaten fruit or drunk 100% fruit juices <1 time/day (47.8% versus 42.1% and 39.5%, respectively), had eaten vegetables <1 time/day (54.8% versus 35.5% and 46.8%, respectively), had drunk a sports drink ≥1 time/day (15.6% versus 9.3% and 11.9%, respectively), and had drunk <3 glasses/day of plain water (54.8% versus 44.2% and 44.2%, respectively). In addition, a higher percentage of Hispanic students than white students had eaten vegetables <1 time/day (46.8% versus 35.3%) and had drunk a sports drink ≥1 time/day (11.9% versus 9.3%), and a higher percentage of black students than white students had not eaten breakfast on all 7 days (21.1% versus 15.3%).

**TABLE 2:** Percentage of high school students who engaged in selected dietary and physical activity behaviors, by sex and race/ethnicity — Youth Risk Behavior Survey, United States, 2019

Variable	Total % (95% CI)	Sex		Race/Ethnicity		
		Female % (95% CI)	Male % (95% CI)	White, non-Hispanic % (95% CI)	Black, non-Hispanic % (95% CI)	Hispanic % (95% CI)
**Dietary behaviors**						
Ate fruit or drank 100% fruit juices <1 time/day*	**41.8 (39.8–43.8)**	43.0 (40.7–45.4)	40.6 (38.2–43.1)	42.1 (39.2–45.1)	47.8^†,§^ (43.6–51.9)	39.5 (36.7–42.3)
Ate vegetables <1 time/day^¶^	**40.7 (38.0–43.4)**	40.4 (37.2–43.6)	41.1 (38.1–44.3)	35.5 (33.2–37.8)	54.8^†,§^ (50.1–59.4)	46.8^†^ (41.8–52.0)
Did not eat breakfast on all 7 days during the 7 days before the survey	**16.7 (15.3–18.1)**	16.7 (15.2–18.3)	16.6 (14.9–18.4)	15.3 (13.9–16.8)	21.1^†^ (17.3–25.6)	16.9 (14.1–20.0)
Drank sugar-sweetened soda or pop ≥1 time/day**	**15.1 (13.1–17.2)**	11.7 (9.9–13.8)	18.2^††^ (15.9–20.8)	15.2 (12.7–18.0)	16.9 (13.5–21.0)	16.1 (13.1–19.6)
Drank a sports drink ≥1 time/day^§§^	**10.6 (9.2–12.3)**	7.1 (5.7–8.8)	14.0^††^ (11.9–16.4)	9.3 (7.7–11.2)	15.6^†,§^ (12.9–18.8)	11.9^†^ (10.2–13.8)
Drank <3 glasses/day of plain water^¶¶^	**44.6 (42.7–46.5)**	44.1 (42.0–46.1)	45.0 (42.3–47.6)	44.2 (41.7–46.7)	54.8^†,§^ (49.0–60.4)	44.2 (41.8–46.7)
**Physical activity behaviors**						
Were physically active for a total of ≥60 minutes/day on all 7 days***	**23.2 (21.9–24.6)**	15.4 (14.2–16.6)	30.9^††^ (28.9–33.1)	25.6 (24.1–27.2)	21.1^†^ (17.6–25.2)	20.9^†^ (18.6–23.5)
Did exercises to strengthen or tone muscles on ≥3 days^†††^	**49.5 (47.6–51.3)**	39.7 (37.2–42.4)	59.0^††^ (56.8–61.0)	50.8 (48.2–53.4)	47.0 (42.7–51.2)	48.1 (44.5–51.9)
Met both aerobic and muscle-strengthening physical activity guidelines^§§§^	**16.5 (14.6–18.6)**	10.1 (8.7–11.6)	23.1^††^ (20.4–26.0)	18.4 (15.8–21.4)	13.4^†^ (9.5–18.4)	16.0 (13.7–18.6)
Went to physical education classes on all 5 days^¶¶¶^	**25.9 (21.5–31.0)**	22.8 (17.9–28.5)	28.9^††^ (24.6–33.7)	24.3 (18.8–30.7)	23.8 (17.4–31.7)	29.9 (24.5–36.0)
Played on ≥1 sports team****	**57.4 (54.3–60.4)**	54.6 (51.1–58.0)	60.2^††^ (56.9–63.4)	62.0 (58.1–65.7)	56.1^†^ (51.4–60.7)	51.6^†^ (46.5–56.6)

**Abbreviation:** CI = confidence interval.

* Such as orange juice, apple juice, or grape juice, not counting punch, Kool-Aid, sports drinks, or other fruit-flavored drinks during the 7 days before the survey.

^†^ Significantly different than white students based on *t*-test analysis (p<0.05).

^§^ Significantly different than Hispanic students based on *t*-test analysis (p<0.05).

^¶^ Green salad, potatoes (not counting French fries, fried potatoes, or potato chips), carrots, or other vegetables during the 7 days before the survey.

** Such as Coke, Pepsi, or Sprite, not counting diet soda or diet pop, during the 7 days before the survey.

^††^ Significantly different than female students based on *t*-test analysis (p<0.05).

^§§^ Such as Gatorade or PowerAde, not counting low-calorie sports drinks such as Propel water or G2, during the 7 days before the survey.

^¶¶^ Counting tap, bottled, and unflavored sparkling water during the 7 days before the survey.

*** Adding up time spent in any kind of physical activity that increased their heart rate and made them breathe hard some of the time during the 7 days before the survey.

^†††^ Such as push-ups, sit-ups, or weightlifting during the 7 days before the survey.

^§§§^ Were physically active for ≥60 minutes/day on all 7 days and did exercises to strengthen or tone muscles on ≥3 of the 7 days before the survey.

^¶¶¶^ In an average week when the student was in school.

**** Counting any teams run by their school or community groups during the 12 months before the survey.

#### Trends

Trend analyses indicated that, during 2009–2019, a significant linear increase occurred in the percentage of students who had eaten fruit or drunk 100% fruit juices <1 time/day overall and among female, male, white, black, and Hispanic students (Table 3). Significant quadratic trends were not identified except among black students. The percentage of black students who had eaten fruit or drunk 100% fruit juices <1 time/day did not change during 2009–2015 and then increased during 2015–2019. During 2017–2019, the percentage of students who had eaten fruit or drunk 100% fruit juices <1 time/day increased among male students and black students.

**TABLE 3:** Percentage of high school students who engaged in selected dietary behaviors, by sex, race/ethnicity, and survey year — Youth Risk Behavior Survey, United States, 2009–2019

Behavior	Prevalence (%)						Linear change*	Quadratic change*	Change during 2017–2019^†^
	2009	2011	2013	2015	2017	2019			
**Ate fruit or drank 100% fruit juices <1 time/day^§^**									
**Total**	**35.2**	**36.0**	**37.4**	**36.7**	**39.2**	**41.8**	**Increased during 2009–2019**	**None**	**None**
Female	37.6	38.4	40.0	37.9	41.8	43.0	Increased during 2009–2019	None	None
Male	33.0	33.9	34.7	35.4	36.7	40.6	Increased during 2009–2019	None	Increased
White, non-Hispanic	34.4	35.8	39.3	37.0	40.4	42.1	Increased during 2009–2019	None	None
Black, non-Hispanic	39.2	36.4	36.5	37.8	39.3	47.8	Increased during 2009–2019	None during 2009–2015 Increased during 2015–2019	Increased
Hispanic	35.6	35.3	35.0	35.9	37.6	39.5	Increased during 2009–2019	None	None
**Ate vegetables <1 time/day^¶^**									
**Total**	**37.3**	**37.7**	**38.5**	**39.0**	**40.6**	**40.7**	**Increased during 2009–2019**	**None**	**None**
Female	38.4	38.4	38.7	40.0	40.7	40.4	None	None	None
Male	36.3	37.2	38.5	38.0	40.6	41.1	Increased during 2009–2019	None	None
White, non-Hispanic	32.7	34.3	35.2	35.8	37.2	35.5	Increased during 2009–2019	None	None
Black, non-Hispanic	48.8	45.7	48.1	47.5	50.6	54.8	Increased during 2009–2019	None during 2009–2015 Increased during 2015–2019	None
Hispanic	45.9	43.6	43.1	43.5	43.9	46.8	None	None	None
**Did not eat breakfast on all 7 days during the 7 days before the survey**									
**Total**	—**	**13.1**	**13.7**	**13.8**	**14.1**	**16.7**	**Increased during 2011–2019**	**—^††^**	**Increased **
Female	—**	13.9	13.8	14.2	14.5	16.7	Increased during 2011–2019	—^††^	Increased
Male	—**	12.3	13.5	13.3	13.6	16.6	Increased during 2011–2019	—^††^	Increased
White, non-Hispanic	—**	12.0	11.5	12.0	12.8	15.3	Increased during 2011–2019	—^††^	Increased
Black, non-Hispanic	—**	16.1	16.0	18.0	15.2	21.1	None	—^††^	Increased
Hispanic	—**	14.4	17.4	14.7	16.0	16.9	None	—^††^	None
**Drank sugar-sweetened soda or pop ≥1 time/day^§§^**									
**Total**	**29.2**	**27.8**	**27.0**	**20.4**	**18.7**	**15.1**	**Decreased during 2009–2019**	**None during 2009–2013** **Decreased during 2013–2019**	**Decreased **
Female	23.3	24.0	24.1	16.4	15.4	11.7	Decreased during 2009–2019	None during 2009–2013 Decreased during 2013–2019	Decreased
Male	34.6	31.4	29.9	24.3	22.3	18.2	Decreased during 2009–2019	None	Decreased
White, non-Hispanic	29.0	28.8	29.0	19.7	19.6	15.2	Decreased during 2009–2019	None	Decreased
Black, non-Hispanic	33.7	28.0	30.2	22.7	21.5	16.9	Decreased during 2009–2019	None	None
Hispanic	28.1	27.0	22.6	21.7	17.0	16.1	Decreased during 2009–2019	None	None
**Drank a sports drink ≥1 time/day^¶¶^**									
**Total**	—**	—**	—**	**13.8**	**12.4**	**10.6**	**Decreased during 2015–2019**	**—^††^**	**None**
Female	—**	—**	—**	8.8	8.2	7.1	None	—^††^	None
Male	—**	—**	—**	18.7	16.9	14.0	Decreased during 2015–2019	—^††^	Decreased
White, non-Hispanic	—**	—**	—**	12.4	10.7	9.3	Decreased during 2015–2019	—^††^	None
Black, non-Hispanic	—**	—**	—**	19.7	21.1	15.6	None	—^††^	Decreased
Hispanic	—**	—**	—**	15.7	13.5	11.9	Decreased during 2015–2019	—^††^	None
Drank <3 glasses/day of plain water***									
**Total**	—**	—**	—**	**50.5**	**48.7**	**44.6**	**Decreased during 2015–2019**	**—^††^**	**Decreased **
Female	—**	—**	—**	51.9	48.8	44.1	Decreased during 2015–2019	—^††^	Decreased
Male	—**	—**	—**	49.0	48.6	45.0	Decreased during 2015–2019	—^††^	Decreased
White, non-Hispanic	—**	—**	—**	50.1	48.8	44.2	Decreased during 2015–2019	—^††^	Decreased
Black, non-Hispanic	—**	—**	—**	60.9	52.7	54.8	None	—^††^	None
Hispanic	—**	—**	—**	49.7	47.5	44.2	Decreased during 2015–2019	—^††^	Decreased

* Based on trend analyses by using a logistic regression model controlling for sex, race/ethnicity, and grade (p<0.05).

^†^ Based on *t*-test analysis (p<0.05).

^§^ Such as orange juice, apple juice, or grape juice, not counting punch, Kool-Aid, sports drinks, or other fruit-flavored drinks, during the 7 days before the survey.

^¶^ Green salad, potatoes (not counting French fries, fried potatoes, or potato chips), carrots, or other vegetables during the 7 days before the survey.

** Data not available. Question not asked in that year.

^††^ Insufficient years of data to assess quadratic trends.

^§§^ Such as Coke, Pepsi, or Sprite, not counting diet soda or diet pop, during the 7 days before the survey.

^¶¶^ Such as Gatorade or PowerAde, not counting low-calorie sports drinks such as Propel water or G2, during the 7 days before the survey.

*** Counting tap, bottled, and unflavored sparkling water during the 7 days before the survey.

During 2009–2019, a significant linear increase occurred in the percentage of students who had eaten vegetables <1 time/day overall and among male, white, and black students. Significant quadratic trends were not identified, except among black students. The percentage of black students who had eaten vegetables <1 time/day did not change during 2009–2015 and then increased during 2015–2019.

During 2011–2019, a significant linear increase occurred in the percentage of students who had not eaten breakfast on all 7 days overall and among female, male, and white students. During 2017–2019, the percentage of students who had not eaten breakfast on all 7 days increased among students overall and among female, male, white, and black students.

**FIGURE 1:**
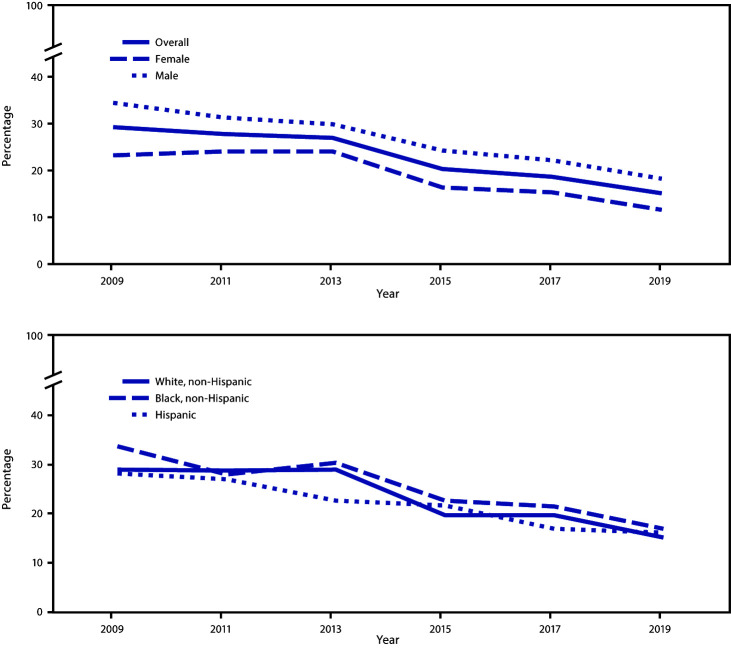
Percentage of high school students who had drunk sugar-sweetened soda or pop ≥1 time per day during the 7 days before the survey, overall and by sex and race/ethnicity* — Youth Risk Behavior Survey, United States, 2009–2019 * During 2009–2019, a significant linear decrease was observed in the percentage of students who had drunk sugar-sweetened soda or pop ≥1 time/day overall and among female, male, white, black, and Hispanic students. Based on trend analyses by using a logistic regression model controlling for sex, race/ethnicity, and grade (p<0.05).

During 2009–2019, a significant linear decrease occurred in the percentage of students who had drunk sugar-sweetened soda or pop ≥1 time/day overall and among female, male, white, black, and Hispanic students (Figure 1). Significant quadratic trends were identified overall and among female students. Overall and among female students, the percentage of students who had drunk sugar-sweetened soda or pop ≥1 time/day did not change during 2009–2013 and then decreased during 2013–2019. During 2017–2019, the percentage of students who had drunk sugar-sweetened soda or pop ≥1 time/day decreased overall and among female, male, and white students.

**FIGURE 2:**
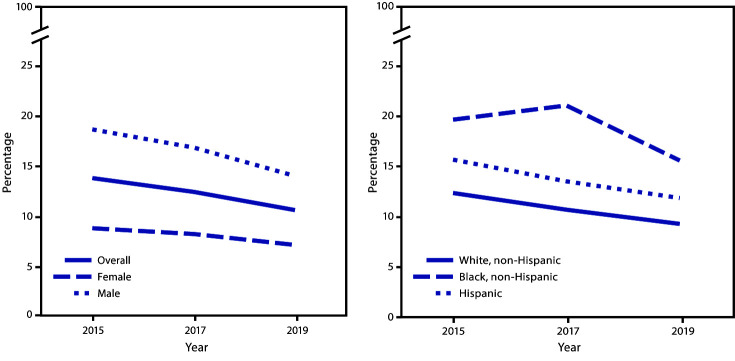
Percentage of high school students who had drunk a sports drink ≥1 time per day during the 7 days before the survey, overall and by sex and race/ethnicity* — Youth Risk Behavior Survey, United States, 2015–2019 * During 2015–2019, a significant linear decrease was observed in the percentage of students who had drunk a sports drink ≥1 time/day overall and among male, white, and Hispanic students. Based on trend analyses by using a logistic regression model controlling for sex, race/ethnicity, and grade (p<0.05).

During 2015–2019, a significant linear decrease occurred in the percentage of students who had drunk a sports drink ≥1 time/day overall and among male, white, and Hispanic students (Figure 2). During 2017–2019, the percentage of students who had drunk a sports drink ≥1 time/day decreased among male students and black students.

During 2015–2019, a significant linear decrease occurred in the percentage of students who had drunk <3 glasses/day of plain water overall and among female, male, white, and Hispanic students. During 2017–2019, the percentage of students who had drunk <3 glasses/day of plain water decreased overall and among female, male, white, and Hispanic students.

### Physical Activity Behaviors

#### Overall

**FIGURE 3:**
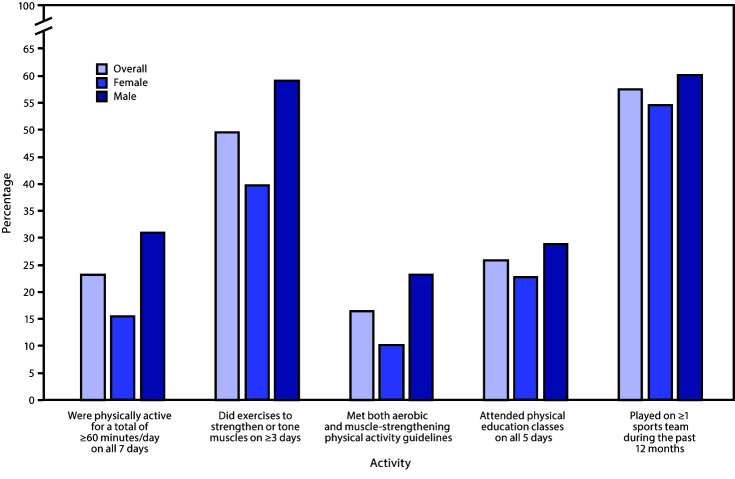
Percentage* of high school students who had engaged in physical activity^†^ and physical education during the 7 days before the survey, overall and by sex^§^ — Youth Risk Behavior Survey, United States, 2019 * Bars represent the percentage of respondents with a “yes” response, overall and by sex. ^†^ The “met both aerobic and muscle-strengthening physical activity guidelines” variable is defined as being physically active for a total of ≥60 minutes/day on all 7 days and doing exercises to strengthen or tone muscles on ≥3 days during the 7 days before the survey (**Source:** U.S. Department of Health and Human Services. Physical activity guidelines for Americans. 2nd ed. Washington, DC: US Department of Health and Human Services; 2018. https://www.hhs.gov/fitness/be-active/physical-activity-guidelines-for-americans/index.html). ^§^ In 2019, a significantly higher percentage of male than female students had been physically active for ≥60 minutes/day on all 7 days during the 7 days before the survey, had exercised to strengthen or tone muscles on ≥3 days during the 7 days before the survey, had met the aerobic and muscle-strengthening physical activity guidelines during the 7 days before the survey, had attended physical education classes on all 5 days in an average school week when the student was in school, and had played on ≥1 sports team during the past 12 months. Based on *t*-test analysis (p<0.05).

In 2019, nationwide, 23.2% of students had been physically active for ≥60 minutes/day on all 7 days; 49.5% had exercised to strengthen or tone their muscles on ≥3 days/week; 16.5% had met both aerobic and muscle-strengthening physical activity guidelines; 25.9% had attended physical education classes on all 5 days in an average school week; and 57.4% had played on ≥1 sports team (Table 2). A higher percentage of male students than female students had been physically active for ≥60 minutes/day on all 7 days (30.9% versus 15.4%), had exercised to strengthen or tone muscles on ≥3 days (59.0% versus 39.7%), had met both aerobic and muscle-strengthening physical activity guidelines (23.1% versus 10.1%), had attended physical education classes on all 5 days in an average school week (28.9% versus 22.8%), and had played on ≥1 sports team (60.2% versus 54.6%) (Figure 3). A higher percentage of white students than black students had been physically active for ≥60 minutes/day on all 7 days (25.6% versus 21.1%), had met both aerobic and muscle-strengthening physical activity guidelines (18.4% versus 13.4%), and had played on ≥1 sports team (62.0% versus 56.1%). In addition, a higher percentage of white students than Hispanic students had been physically active for ≥60 minutes/day on all 7 days (25.6% versus 20.9%) and had played on ≥1 sports team (62.0% versus 51.6%) (Table 2).

#### Trends

During 2011–2019, a significant linear decrease occurred in the percentage of students who had been physically active for ≥60 minutes/day on all 7 days overall and among female, male, white, black, and Hispanic students (Table 4). During 2017–2019, the percentage of students who had been physically active for ≥60 minutes/day on all 7 days decreased overall and among male students and Hispanic students.

**TABLE 4:** Percentage of high school students who engaged in selected physical activity behaviors, by sex, race/ethnicity, and survey year — Youth Risk Behavior Survey, United States, 2009–2019

Behavior	Prevalence (%)						Linear change*	Quadratic change*	Change during 2017–2019^†^
	2009	2011	2013	2015	2017	2019			
**Were physically active for a total of ≥60 minutes/day on all 7 days^§^**									
**Total**	**—** ^¶^	**28.7**	**27.1**	**27.1**	**26.1**	**23.2**	**Decreased during 2011–2019**	—**	**Decreased during** **2017–2019**
Female	—^¶^	18.5	17.7	17.7	17.5	15.4	Decreased during 2011–2019	—**	None
Male	—^¶^	38.3	36.6	36.0	35.3	30.9	Decreased during 2011–2019	—**	Decreased during 2017–2019
White, non-Hispanic	—^¶^	30.4	28.2	29.0	27.1	25.6	Decreased during 2011–2019	—**	None
Black, non-Hispanic	—^¶^	26.0	26.3	24.2	24.5	21.1	Decreased during 2011–2019	—**	None
Hispanic	—^¶^	26.5	25.5	24.6	25.8	20.9	Decreased during 2011–2019	—**	Decreased during 2017–2019
**Did exercises to strengthen or tone muscles on ≥3 days** ^††^									
**Total**	**—^¶^**	**55.6**	**51.7**	**53.4**	**51.1**	**49.5**	**Decreased during 2011–2019**	—**	**None**
Female	—^¶^	43.8	41.6	42.7	40.8	39.7	None	—**	None
Male	—^¶^	66.7	61.8	63.7	62.1	59.0	Decreased during 2011–2019	—**	None
White, non-Hispanic	—^¶^	55.7	52.4	54.5	50.6	50.8	Decreased during 2011–2019	—**	None
Black, non-Hispanic	—^¶^	54.0	48.8	52.3	51.0	47.0	Decreased during 2011–2019	—**	None
Hispanic	—^¶^	56.6	53.3	52.4	52.3	48.1	Decreased during 2011–2019	—**	None
**Met guidelines for aerobic and muscle-strengthening physical activity** ^§§^									
**Total**	**—^¶^**	**21.9**	**21.6**	**20.5**	**20.0**	**16.5**	**Decreased during 2011–2019**	—**	**None**
Female	—^¶^	12.7	13.0	12.2	12.1	10.1	Decreased during 2011–2019	—**	None
Male	—^¶^	30.7	30.3	28.6	28.5	23.1	Decreased during 2011–2019	—**	Decreased during 2017–2019
White, non-Hispanic	—^¶^	23.9	22.6	22.7	20.8	18.4	Decreased during 2011–2019	—**	None
Black, non-Hispanic	—^¶^	18.4	20.6	15.7	17.7	13.4	Decreased during 2011–2019	—**	None
Hispanic	—^¶^	18.9	20.5	18.7	20.0	16.0	None	—**	Decreased during 2017–2019
**Went to physical education classes on all 5 days** ^¶¶^									
**Total**	**33.3**	**31.5**	**29.4**	**29.8**	**29.9**	**25.9**	**None**	**None**	**None**
Female	31.9	27.2	24.0	25.5	25.3	22.8	Decreased during 2009–2019	None	None
Male	34.6	35.5	34.9	33.8	34.7	28.9	None	None	None
White, non-Hispanic	30.6	33.0	27.1	25.4	27.2	24.3	None	None	None
Black, non-Hispanic	37.0	27.6	26.6	35.8	28.5	23.8	None	None	None
Hispanic	40.5	30.0	37.7	37.7	37.4	29.9	None	None	None
Played on ≥1 sports team***									
**Total**	**58.3**	**58.4**	**54.0**	**57.6**	**54.3**	**57.4**	**None**	**None**	**None**
Female	52.3	52.6	48.5	53.0	49.3	54.6	None	None	None
Male	63.8	64.0	59.6	62.2	59.7	60.2	None	None	None
White, non-Hispanic	61.1	60.9	55.2	62.4	54.5	62.0	None	None	None
Black, non-Hispanic	57.3	57.0	55.2	57.6	59.1	56.1	None	None	None
Hispanic	53.2	54.1	51.2	48.5	52.2	51.6	None	None	None

* Based on trend analyses by using a logistic regression model controlling for sex, race/ethnicity, and grade (p<0.05).

^†^ Based on *t*-test analysis (p<0.05).

^§^ Adding up time spent in any kind of physical activity that increased their heart rate and made them breathe hard some of the time during the 7 days before the survey.

^¶^ Data not available. Question not asked in that year.

** Insufficient years of data to assess quadratic trends.

^††^ Such as push-ups, sit-ups, or weightlifting during the 7 days before the survey.

^§§^ Were physically active for ≥60 minutes/day on all 7 days and did exercises to strengthen or tone muscles on ≥3 of the 7 days before the survey.

^¶¶^ In an average week when the student was in school.

*** Counting any teams run by their school or community groups during the 12 months before the survey.

During 2011–2019, a significant linear decrease occurred in the percentage of students who had exercised to strengthen or tone their muscles on ≥3 days/week overall and among male, white, black, and Hispanic students. During 2017–2019, no significant changes occurred in the percentage of students who had exercised to strengthen or tone their muscles on ≥3 days/week overall or among the sex or racial/ethnic groups.

During 2011–2019, a significant linear decrease occurred in the percentage of students who had met both aerobic and muscle-strengthening physical activity guidelines overall and among female, male, white, and black students. During 2017–2019, the percentage of students who had met both aerobic and muscle-strengthening physical activity guidelines did not significantly change overall but decreased among male students and Hispanic students.

During 2009–2019 and during 2017–2019, no significant linear changes occurred in the percentage of students who had attended physical education classes on all 5 days in an average school week or had played on ≥1 sports team overall or among the sex and racial/ethnic groups, except among female students. Among female students, a significant linear decrease occurred in the percentage who had attended physical education classes on all 5 days in an average school week.

## Discussion

With the exception of decreases in the percentages of students who had consumed soda ≥1 time/day, sports drinks ≥1 time/day, and <3 glasses/day of plain water, high school students’ dietary and physical activity behaviors have not improved during the previous 10 years and, in certain cases, have worsened. This is cause for concern because healthy dietary and physical activity behaviors are important for growth and development, academic outcomes, and prevention of chronic diseases, including type 2 diabetes, heart disease, hypertension, and obesity (*1**,**7*). Recent data demonstrate that approximately one in five adolescents have prediabetes, which increases the risk for type 2 diabetes and cardiovascular diseases (*8*). In addition, data from the National Health and Nutrition Examination Survey reveal that, in the United States during 2007–2008, approximately 18.1% of youths aged 12–19 years had obesity and this increased to 20.6% during 2015–2016 (*9*). In this analysis, in which differences by race/ethnicity exist, black and Hispanic high school students have poorer dietary and physical activity behaviors, compared with white high school students. These findings also indicate that male students have poorer dietary behaviors but better physical activity behaviors than do female students. Addressing dietary and physical activity behaviors can benefit all students and is especially important for those with increased risk for chronic diseases (e.g., students from low-income families and racial/ethnic minorities).

### Dietary Behaviors

No improvements occurred in fruit or vegetable consumption during 2009–2019 and, in many cases, have worsened. Overall, consumption of fruits and vegetables remained low in 2019. For example, four of 10 high school students had eaten fruit or drunk 100% fruit juices <1 time/day. Similarly, four of 10 had eaten vegetables <1 time/day. Although the prevalence of having eaten fruit or drunk 100% fruit juice <1 time/day and having eaten vegetables <1 time/day is similar for male students and female students, recommended daily intakes differ by age and sex. Females and males aged 14–18 years need 1.5 cups and 2 cups, respectively, of fruits, and 2.5 cups and 3 cups, respectively, of vegetables (https://www.choosemyplate.gov/resources/MyPlatePlan). Although YRBS measures frequency of intake and not the amount consumed, children and adolescents who meet the recommended amounts typically consume fruits and vegetables multiple times throughout the day (*10*); therefore, consuming fruits or vegetables <1 time/day is likely insufficient. Strategies that encourage adolescents to increase the quantity of fruits and vegetables each time they consume them are likely needed to help them meet the daily recommendations (*10*). For example, schools can offer students multiple fruit and vegetable choices each day through school meal programs, including through grab-and-go salads (*11*).

Sugar-sweetened beverages (SSBs) are the primary source of added sugars in U.S. youths’ diets (*1*). Frequently drinking SSBs is associated with health conditions, including obesity, type 2 diabetes, heart disease, and tooth decay (*12*). Alternatively, drinking enough water every day is good for overall health and is associated with higher Healthy Eating Index scores among adolescents (*13*). (More information about the Healthy Eating Index is available at https://www.fns.usda.gov/resource/healthy-eating-index-hei.) YRBS asks about two specific types of SSBs, soda or pop and sports drinks. This study identified substantial decreases in the percentage of students who had drunk soda or pop ≥1 time/day overall and among all sex and racial/ethnic groups. In addition, decreases occurred in the percentage of students who had drunk a sports drink ≥1 time/day overall and among female, white, and Hispanic students. Despite these improvements in soda and sports drink consumption, consumption of these beverages is common. Differences also existed by sex and race/ethnicity. Similar to this study, previous studies reported that SSB intake was higher among males than among females (*14*) and among black and Hispanic adolescents than among white adolescents (*15*). One possible explanation for the differences between racial/ethnic groups is that beverage companies disproportionately market SSBs to black and Hispanic youths (*16*).

During the 2014–15 school year, the Smart Snacks in School nutrition standards were implemented, which decreased students’ access to SSBs at school. (More information about the Smart Snacks in School nutrition standards is available at https://www.gpo.gov/fdsys/pkg/FR-2013-06-28/pdf/2013-15249.pdf.) Additional policy and educational approaches (e.g., health education classes or communitywide campaigns) might help further reduce SSB access in schools and other settings and help adolescents choose healthier beverage options, including plain water.

### Physical Activity Behaviors

Overall, prevalence of health-promoting physical activity behaviors was low in 2019 and either decreased or did not change during the previous 10 years. *Healthy People 2020* monitors four of the five physical activity behaviors included in this study (https://www.healthypeople.gov/), and these behaviors will continue to be monitored with *Healthy People 2030*. *Healthy People 2020* objective PA-3 aims to increase the proportion of adolescents who meet federal physical activity guidelines for aerobic physical activity to ≥31.6% (PA-3.1), muscle-strengthening activity to ≥61.2% (PA-3.2), and both aerobic physical activity and muscle-strengthening activity to ≥24.1% (PA-3.3). The proportions of students meeting the aerobic, muscle-strengthening, or both guidelines decreased during 2011–2019, and 2019 data indicate that adolescents continue to fall short of achieving these targets.

One of the *Healthy People 2020* objectives (PA-5) is to increase the proportion of adolescents who participate in daily school physical education to ≥36.6%. Given no increase in this behavior during 2009–2019 and that only 25.9% of high school students attended daily physical education class during 2019, the target for this objective is unlikely to be met in 2020. Students can accumulate approximately 40% of their daily physical activity through participation in physical education (*17*), demonstrating that physical education at school is an effective strategy for helping high school students meet the federal physical activity guidelines. During 2015–2016, although the majority of U.S. states required public high schools to provide physical education, few states mandated a time requirement for high school students, and many states permitted students to substitute other activities for their physical education requirement. (More information about the status of physical education in the United States is available at https://www.shapeamerica.org/MemberPortal/SHAPE_Sign_I.aspx?WebsiteKey=c03f2b51-3ee7-46fa-b587-de18213dcae5&LoginRedirect=true&returnurl=%2fadvocacy%2fson%2f.)

The 2019 release of the National Youth Sports Strategy highlighted youth sports participation for its physical activity, psychosocial, and academic achievement benefits. (More information about the National Youth Sports Strategy is available at https://health.gov/our-work/physical-activity/national-youth-sports-strategy.) Despite these benefits, only 57.4% of high school students reported participating in sports. The National Survey of Children’s Health also assesses participation in youth sports, with similar estimates to YRBS for youths aged 14–17 years (*3*).

Across all the physical activity behaviors, a higher percentage of males than females met aerobic, muscle-strengthening, or both guidelines, participated in daily physical education, and played on ≥1 sports team. These differences might be caused by gender stereotypes, self-efficacy, self-consciousness, or social influences (*18*). When overcoming barriers to physical activity, particularly for adolescent females, strategies that span the Social-Ecological Model by addressing individual, interpersonal, organizational, community, and societal components might need to be considered.

### Addressing Dietary and Physical Activity Behaviors

Improving dietary and physical activity behaviors among adolescents requires efforts across multiple settings. For example, schools can implement policies and practices (e.g., local school wellness policies) (https://www.fns.usda.gov/tn/local-school-wellness-policy) that support healthy eating and physical activity, including ensuring the following: 1) that foods and beverages sold during the school day meet Smart Snacks in School nutrition standards, 2) that school meals are appealing and include menu items that students enjoy, and 3) that students have access to free drinking water during the school day (*11*). Schools can also help students meet the federal physical activity guidelines by providing physical activity opportunities before, during, and after the school day. This can be achieved by developing, implementing, and evaluating a comprehensive school physical activity program, which serves as a national framework for physical education and physical activity in schools. (More guidance on comprehensive school physical activity programs is available at https://www.cdc.gov/healthyschools/physicalactivity/pdf/13_242620-A_CSPAP_SchoolPhysActivityPrograms_Final_508_12192013.pdf.)

Health education is another way that schools can help students develop the knowledge and skills needed for making health-enhancing decisions. These school efforts can be addressed and coordinated through the Whole School, Whole Community, Whole Child Model, which highlights the interconnectedness of multiple health behaviors and outcomes and promotes collaboration among diverse partners, including mental health professionals, school leaders, school nurses, physical and health educators, and parents for promoting health and well-being for all students. (More information about the Whole School, Whole Community, Whole Child approach is available at https://www.cdc.gov/healthyschools/wscc/index.htm.) 

Community members and parents can reinforce the messages promoted within the school and can participate on the school wellness or school health teams that are addressing healthy eating, physical education, and physical activity. (More information about parent engagement in school health is available at https://www.cdc.gov/healthyyouth/protective/pdf/parent_engagement_strategies.pdf.) In addition, parents and community members can engage in physical activity with adolescents, provide social supports for adolescents that increase physical activity while decreasing sedentary behaviors, and make choices that support healthy eating.

Community-based interventions that address healthy eating and physical activity through policy and environmental changes can improve dietary and physical activity behaviors and weight-status outcomes among youths (*19*–*21*). These kinds of community-based approaches often adopt multiple strategies, including providing information (e.g., messaging campaigns and healthy recipe demonstrations), providing incentives, and improving access to opportunities for practicing healthy behaviors through policy and systems changes. Having multiple activities that target specific behaviors and using a mix of behavioral change strategies appear to be important for making health behavior changes (*19*). Community-based interventions that also include the school setting are more effective in influencing outcomes among youths than interventions that occur only in the community (*20*).

## Limitations

General limitations for the YRBS are available in the overview report of this supplement (*5*). The findings in this report are subject to at least one additional limitation. Certain questions about dietary behaviors (e.g., fruit consumption) ask about frequency rather than portion size; therefore, these data cannot directly determine whether students are meeting specific recommendations for age and sex (*22*). 

## Conclusion

Because of the limited progress in increasing the prevalence of healthy dietary and physical activity behaviors among U.S. high school students, multicomponent approaches, including policy and environmental changes and opportunities for adolescents to learn about and practice making healthy choices, are needed to facilitate healthy dietary and physical activity patterns. Schools, communities, and families can work together in creating healthy environments where adolescents thrive.
